# Optical Absorption of the Antitrypanocidal Drug Benznidazole in Water

**DOI:** 10.3390/molecules19044145

**Published:** 2014-04-02

**Authors:** Eveline M. Bezerra, João R. Bezerra-Neto, Francisco A. M. Sales, Ricardo P. dos Santos, Alice M. C. Martins, Pedro de Lima-Neto, Ewerton W. S. Caetano, Eudenilson L. Albuquerque, Valder N. Freire

**Affiliations:** 1 Departamento de Análises Clínicas e Toxicológicas, Universidade Federal do Ceará, Campus do Porangabuçu, Fortaleza 60430-270, CE, Brazil; 2 Departamento de Química Analítica e Fisico-Química, Universidade Federal do Ceará, Campus do Pici, Fortaleza 60440-900, CE, Brazil; 3 Departamento de Física, Universidade Federal do Ceará, Campus do Pici, Caixa Postal 6030, Fortaleza 60440-900, CE, Brazil; 4 Instituto Federal de Educação, Ciência e Tecnologia do Ceará, Av. Treze de Maio, 2081, Benfica, Fortaleza 60040-531, CE, Brazil; 5 Departamento de Biofísica e Farmacologia, Universidade Federal do Rio Grande do Norte, Natal-RN 59072-970, Brazil

**Keywords:** benznidazole, antitrypanocidal drug, optical absorption, TD-DFT calculations, aqueous environment

## Abstract

UV-vis optical absorption spectra of the antitrypanocidal drug benznidazole solvated in water were measured for various concentrations. The spectra show a prominent peak around 3.80 eV, while deconvolution of the UV-vis optical absorption spectra revealed six bands centered at 3.60, 3.83, 4.15, 4.99, 5.60, and 5.76 eV. Benznidazole electronic transitions were obtained after density functional theory (DFT) calculations within the polarized continuum (PCM) model for water solvation. Molecular geometry optimizations were carried out, and the measured absorption peaks were related to specific molecular orbital transitions obtained within the time dependent DFT (TD-DFT) with excellent agreement between theory and experiment.

## 1. Introduction

Nitroheterocycle benznidazole (BZN) and nifurtimox (NFX) continue to be, about 30 and 40 years after initial experimental assays, respectively, the most used drugs to treat Chagas disease [1]. Chagas is lifelong and it is caused by the protozoan parasite Trypanosoma cruzi, being recognized by the World Health Organization [2] as one of the thirteen most important neglected diseases worldwide. Both BZN and NFX are unable to provide a definitive cure for Chagas due to several limitations, such as strong adverse events, and both medicines are more effective only in the early stages of the disease but not in its chronic phase. Resistance of several Chagas strains against treatment was observed as well [3], and the poor solubility of these drugs in water is a serious difficulty for their practical use [4]. We focus on BZN in this work as it is the preferred choice due to the severe NFX gastrointestinal and neurological side effects. Traditional pharmaceutical [4] as well as nanotechnological approaches [5,6] are being developed to enhance the BZN efficacy, improving its potency, reducing its toxicity, and increasing its solubility, which demands a deeper understanding of its molecular properties and protein targets to correlate the BZN structural characteristics with its trypanosomacidal effect.

Reviewed pediatric trials showed that antitrypanosomal drug treatment in children with chronic T. cruzi infection was accepted as the care standard throughout Latin America by the late 1990s [7], followed by a crescent trend to offer treatment to older patients in recent years. Most experts now believe that the majority of patients up to 50 years of age who have chronic T. cruzi infection [7], including those without symptoms and those with early manifestations of cardiomyopathy, must receive antitrypanosomal treatment. However, the recommended dosage of benznidazole is given in tablet form, which is inadequate for children, neonates and the elderly [8]. The development of liquid benznidazole formulations, on the other hand, needs more physical-chemical data to allow for their characterization. Since liquid environments (mainly water) impose serious problems for the vibrational (principally the IR) characterization of solvated molecules, one has to rely on Nuclear Magnetic Resonance (NMR) and optical absorption spectroscopies to obtain details of the aqueous form of benznidazole. In particular, the optical absorption of solvated benznidazole could be very helpful as a characterization technique in a pharmaceutical production line.

**Figure 1.**
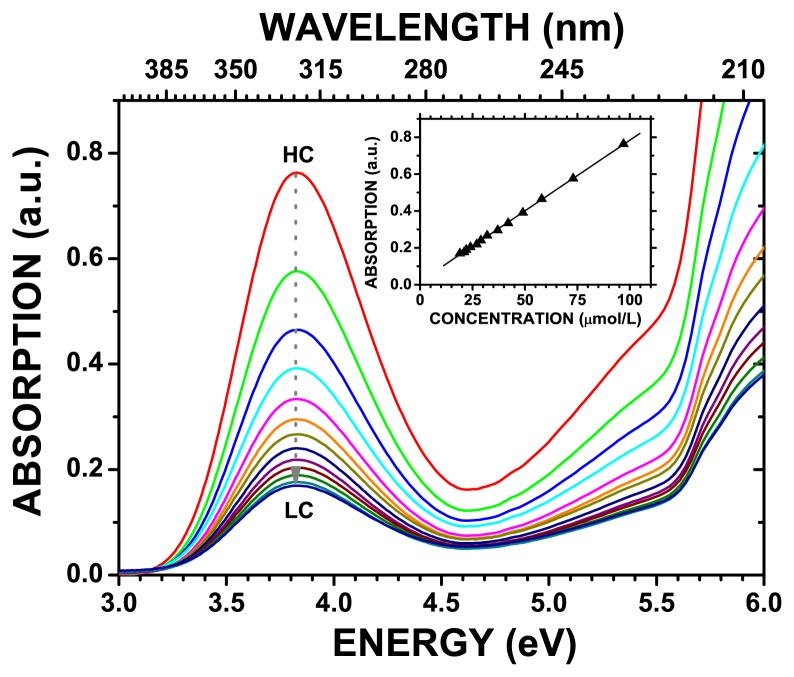
UV-vis optical absorption spectra of benznidazole in water for concentrations of 146.0 (HC), 97.3, 73.0, 58.4, 48.7, 41.7, 36.5, 32.4, 29.2, 26.5, 24.3, 22.5, 20.9, and 19.5 (LC) μmol/L.

Despite the Chagas disease recognition as a relevant social and economic problem in several Latin America countries [9], it is surprising that very few data on the BZN properties are available in the vibrational [8] and in the UV-vis optical optical absorption spectroscopy fields [10,11]. As a matter of fact, even the benznidazole conformation in the solid state was resolved only recently through X-ray diffraction measurements carried out by Soares-Sobrinho *et al.* [12]. The benznidazole conformation in the crystalline state was described in terms of the relative orientation of three planar fragments, *i.e.*, the groups imidazol, benzyl, and acetamide (see Figure 1 below). On the other hand, preliminary ^1^H Nuclear Magnetic Resonance (NMR) and vibrational (infrared and Raman) spectroscopic data in the 500–3500 cm^−1^ range were published only two years ago by Soares-Sobrinho *et al.* [8]. For the latter, vibrational band assignments were performed without the aid of theoretical computations of the benznidazole molecular properties. It is worth to mention that Silva *et al.* [11] have optimized the BZN structure using density functional theory (DFT) calculations without publishing their structural data. They have also measured the UV-vis optical absorption spectra of BZN in water, ethanol and acetone, and have argued that their experimental electronic absorption spectra data are in agreement with their TD-DFT computations [11], but without exhibiting any plot depicting their measured UV-vis and simulated spectra, and not giving a completely adequate description of their experimental and theoretical procedures.

It is quite regrettable that improved experimental or theoretical information on benznidazole structural conformers in vacuum or solvated are still missing nowadays. Our research group has recently measured the benznidazole vibrational properties in vacuum and at room temperature to allow for an improved DFT-based assignment of its vibrational modes. Through a scan of a large set of structural configurations, we have found two benznidazole conformers with very close total energy values: BZN1, which has the smallest energy, has the benzene and imidazole rings in opposite sites relative to the acetamide group, its structure resembling that of the crystal state [12]; the second smaller energy conformation, BZN2, has the benzene and imidazole rings facing each other. A comparison between the solid-state measured and the BZN1 and BZN2 calculated spectra at 1350–1450 cm^−1^ allowed us to confirm that the benznidazole samples used in the IR and Raman measurements have only the BNZ1 structure of the crystal. Besides, we performed a molecular docking study of both conformers in the sterol 14*α*-demethylase CYP51 enzyme demonstrating that BZN1 has the best pose, suggesting not only the benznidazole mode of action at the proteic level, but also a smaller pharmacological activity of the BZN2 conformation (in comparison to BZN1) with respect to their role as an anti-fungal azole inhibitor of CYP51 for the treatment of Chagas disease [13].

In this work we characterize the antitrypanocidal drug benznidazole in water through optical absorptions measurements and *ab initio* molecular computing, with both DFT and TD-DFT calculations being performed for an improved understanding of the experimental UV-vis optical absorption data. The measurements were performed for several BZN concentrations in water, with the obtained spectra showing a characteristic broad absorption peak around 3.80 eV. The spectra in the 3.0–6.0 eV energy range were deconvoluted into six Gaussian absorption components. A computational scan was performed to find out the possible benznidazole conformers in the framework of the polarized continuum model (PCM) to take into account the water environment. Besides, the DFT electronic structure of the lowest energy BZN conformer in water is calculated, as well as its HOMO, LUMO energy levels and orbitals, which are related to the main optical absorption transitions. Finally, an improved description of the optical absorption spectra through TD-DFT calculations is presented to explain the measured benznidazole UV-vis optical absorption data, with the most relevant optical transitions strongly correlated to the deconvoluted Gaussian peaks.

## 2. Results and Discussion

### 2.1. Samples Preparation and Measurements

**Figure 2.**
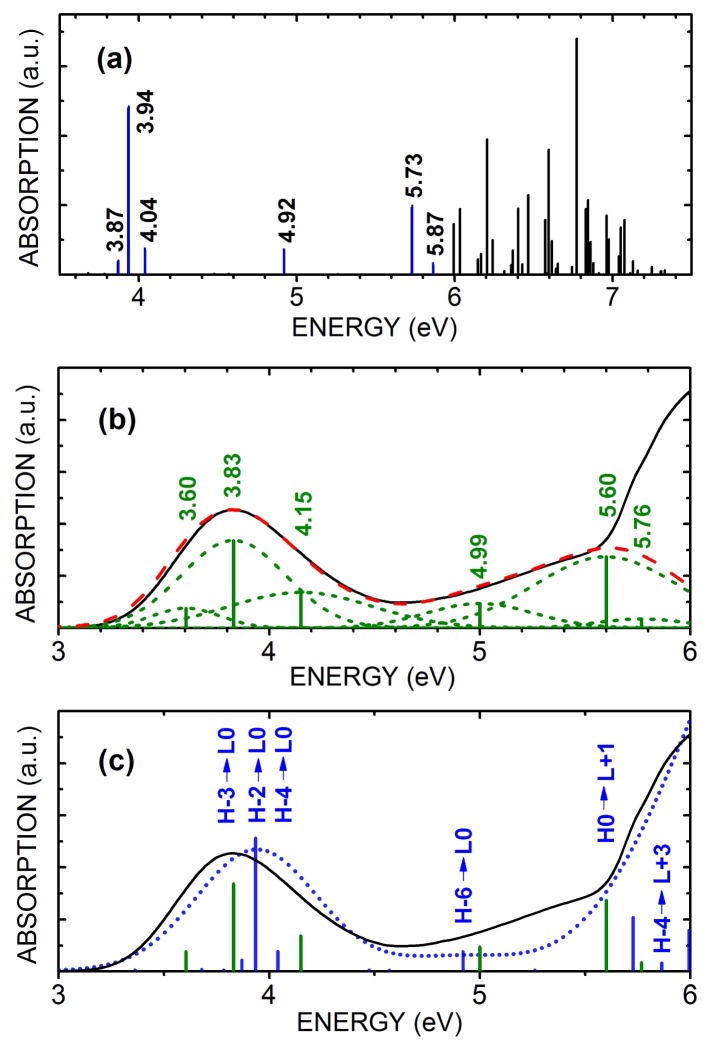
(**a**) TD-DFT calculated UV-vis optical absorption of benznidazole in water (PCM model) at the highest concentration 146.0 μmol/L (HC); (**b**) deconvolution of the the measured UV-vis optical absorption of benznidazole in water (PCM model) for the HC case using six Gaussian curves (dashed lines), with corresponding mean energies indicated by vertical bars; (**c**) a comparison between the measured UV-vis optical absorption of benznidazole in water (PCM model) for HC (solid black line) and the TD-DFT calculated spectrum (short dotted blue line). The mean energy positions for each Gaussian peak fitting the measured UV-vis spectrum are indicated by green vertical bars, while the main TD-DFT transition energies are depicted using blue vertical bars. For the latter, the highest relevant HOMO and LUMO orbitals involved in the excitations are assigned.

99% pure benznidazole powder was kindly supplied by the Pernambuco State Pharmaceutical Laboratory (LAFEPE), in Recife-PE, Brazil, and probed by X-ray scattering (data not presented here) to ensure that the microcrystals in the powder were monoclinic *P2*_1_. The UV-vis spectra of the benznidazole powder dissolved in milli-Q water were measured with the NIR spectrophotometer Varian Cary 5000 UV-visible (Varian Inc., Palo Alto, CA, USA), spectral window 200–800 nm and optical path length 1000 cm. Water dissolved benznidazole spectral data are expressed as absorbance (A) versus wavelength (in nm), as well as energy (in eV). All dilutions were made from an initial high concentration (HC, 146.0 μmol/L) to a final low concentration (LC, 19.5 μmol/L) of benznidazole in water, with intermediary concentrations of 97.3, 73.0, 58.4, 48.7, 41.7, 36.5, 32.4, 29.2, 26.5, 24.3, 22.5, and 20.9 μmol/L—see Figure 1. To determine the molar absorptivity coefficient (*ε*) at 324 nm, a linear regression was performed using the optical absorption value measured for each concentration. Finally, spectral deconvolution of the HC benznidazole was performed employing six Gaussian fitting curves within the 3–6 eV range—see Figure 2b.

### 2.2. Computational Details

The atomic geometry of the isolated uncharged benznidazole (a neutral molecule at physiological pH) was optimized in water through density functional theory (DFT) calculations within the polarizable continuum model (PCM) using the Gaussian03 code [14]. The hybrid B3LYP exchange-correlation functional and the 6-311+G(d,p) basis set were employed, and the quality of the minimization procedure was checked by verifying the absence of vibrational normal modes with imaginary frequencies. Structural optimization was achieved after the following convergence thresholds were satisfied: maximum force smaller than 1.5 × 10^−5^ Ha/Å^−1^, RMS force smaller than 1.0 × 10^-5^ Ha/Å^−1^, self-consistent field energy variation smaller than 10^−7^ Ha, and maximum atomic displacement smaller than 6 × 10^−5^ Å. Excited states were calculated using the time-dependent density functional theory formalism, which can be considered an exact reformulation of time-dependent quantum mechanics where particle states are replaced by density states [15,16]. In this approach, a set of modified Kohn-Sham equations describe the evolution of the electron density using non-interacting electrons in an effective exchange-correlation time-dependent functional. Excitation energies can be obtained by analyzing the response of the system to small time-dependent perturbations, such as photons (linear response theory formalism).

The time-dependent density functional theory (TD-DFT) calculations were performed with the Gaussian03 code also. The fifty lowest singlet-singlet vertical electronic excitations were requested keeping the same functional, basis set and PCM solvation of the geometry optimization step. TD-DFT is able to reproduce very satisfactorily the optical absorption experimental band shape—see review papers about TD-DFT [15,17]. Vertical TD-DFT calculations typically yield the transition energies, the oscillator strengths as well as the molecular orbital (MO) compositions corresponding to each excited-state, as explicitly stated by Adamo and Jacquemin in their tutorial review [17]. Whilst the former are self-explanatory, the second can be related to experimental molar absorption coefficients, and thus to the intensities of the measured absorption bands. The last information allows a chemical interpretation of the results that is, one could relate the electronically excite states to a transition between given MO [17].

## 3. Experimental

The measured 3.0–6.0 eV UV-vis optical absorption spectra of benznidazole in water for concentrations in the 19.5–146.0 μmol/L range are depicted in Figure 1, where it is shown that the highest (smallest) absorption occurs for the 146.0 (19.5) μmol/L benznizadole concentration. One can observe a broad band centered around 3.8 eV whose intensity gradually diminishes when the benznidazole concentration in water becomes smaller. This decrease is practically linear, as shown in the inset of Figure 1, being fitted by the equation *y* = *b* + *a* × *x* = 0.0177 + 0.0077 × *x*, where *y* is the intensity of the optical absorption and *x* the benznidazole concentration in water (μmol/L). The *a* and *b* parameters have estimated uncertainties Δ*a* = 0.0024 and Δ*b* = 5.2*E* − 5, with statistical deviation of 0.9997. The deconvolution of the UV-vis absorption spectra of benznidazole in water with Gaussian curves gives rise to peaks centered at energies which can be compared with the DFT calculated HOMO-LUMO transition energies, as well as with the TD-DFT excitations with more intense oscillator strengths.

**Figure 3.**
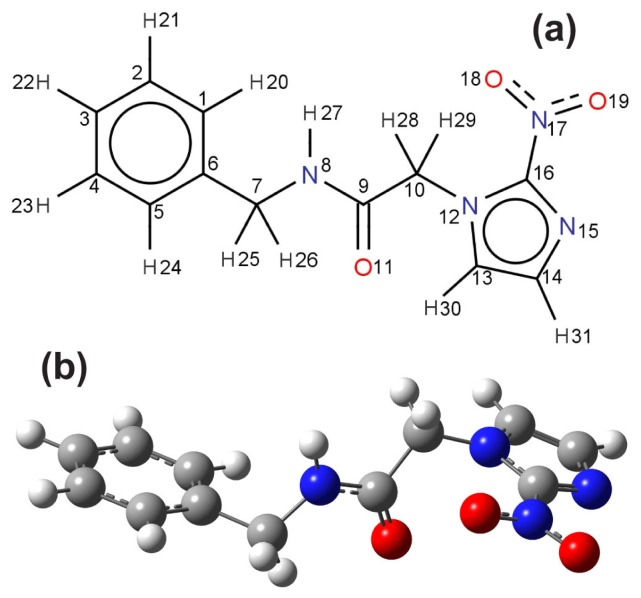
(**a**) Planar representation of benznidazole and atom labels; (**b**) Geometry of the DFT calculated lowest energy conformer of benznidazole using a PCM aqueous solvation model.

The planar molecular structure of benzonidazole and its atomic labels are shown in Figure 3a. As in the vacuum case, a scan of a multitude of benznidazole conformations in water (described within the scope of the PCM model) suggests the existence of two conformers with formation energies differing by less than *kT* at 300 K. However, only the lowest energy conformer was considered in this work for the UV-vis optical absorption calculations, which is depicted in Figure 3b. Tables S1–S3 of the Supplementary Material show that the bond lengths, angles and dihedral angles of the X-ray benznidazole crystal structure are not very distinct from the DFT-calculated corresponding values for the water solvated benznidazole conformer we have considered for the TD-DFT UV-vis optical absorption calculations.

Figure 2a depicts the TD-DFT calculated excited states oscillator strengths for benznidazole within the water PCM model. There is a cluster of optically active transitions around 3.94 eV, a single active transition at 4.921 eV, and a broad set of optical transitions within the 5.0–7.5 eV range. The optical transitions around 3.94 eV involves eight molecular states, according with Table 1, but only three terms contribute significantly to the oscillator strength: H-3→L0, with 67.4% of the 0.0191 oscillator strength and transition energy of 3.87 eV; H-2→L0, with 85.5% of the 0.0349 oscillator strength and transition energy of 3.94 eV; and H-4→L0, with 63.9% of the 0.0349 oscillator strength and transition energy of 4.04 eV The single state at 4.92 eV has contributions from two transitions, the most important being H-6→L0, with 92.5% of the 0.0343 oscillator strength and transition energy of 4.92 eV The most active states in the 5.0–6.0 eV range have two significant transitions: H0→L+1, with 63.7% of the 0.0963 oscillator strength and transition energy of 5.73 eV, and H-4→L+3, with 18.2% of the 0.0138 oscillator strength and transition energy of 5.87 eV.

The high concentration (HC) water solvated benznidazole has its optical absorption spectrum depicted in Figure 2b. Six Gaussian peaks were used to fit the spectral curve in the 3.0–6.0 eV range, with the best adjust being observed by centering the peaks at 3.60, 3.83, 4.15, 4.99, 5.60, and 5.76 eV. The curve resulting from the addition of the Gaussian curves exhibited a 0.999 mean error deviation from experimental data, and it is shown in Figure 2b as a dashed red line; the theory-experiment shifts obtained for the TD-DFT calculated transition energies are −6.9%, −2.2%, +3.5%, +1.4%, −0.23%, and −1.9%. Thus, the TD-DFT calculated transition energies are in remarkable agreement with those obtained from the Gaussian deconvolution of the measured UV-vis optical absorption of the water dissolved antitrypanocidal drug benznidazole at the 146.0 μmol/L concentration. This is easily seen by contrasting the experimental (solid black) and theoretical (short-dotted blue) curves depicted in Figure 2c, together with the center-peak energy values of the Gaussian curves fitted to experiment in Figure 2b (green vertical bars). In Figure 2c, one can see also the six most important TD-DFT calculated contributions for the excited states involved in the optically active transitions (blue vertical bars).

**Figure 4.**
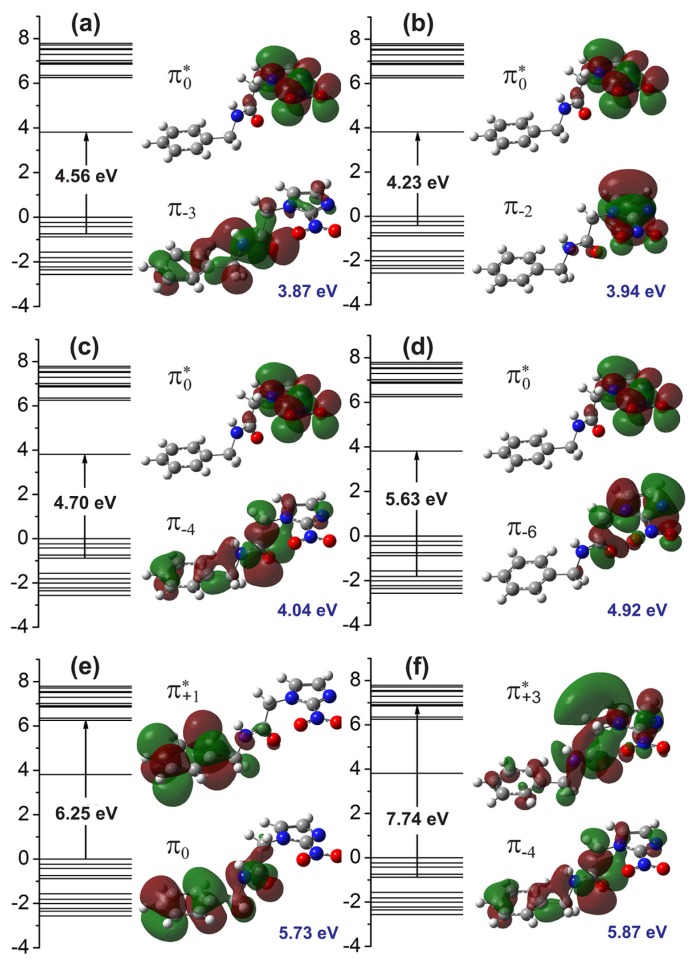
(**a–f**) The six most important calculated energy transitions between the HOMO and LUMO orbitals of benznidazole in water in order of increasing energy. The main electronic configurations behind each transition are shown.

Figure 4 presents the molecular orbitals involved in the main optical absorption bands. For the 3.87 eV excitation energy (Figure 4a), we have a strong contribution from the $\pi_{-3}\to \pi_{0}^{*}$ transition (4.56 eV), where the *π*_−3_ orbital is delocalized across the benzene and acetamide regions, while the $\pi_{0}^{*}$ orbital (LUMO) is localized at the imidazole ring. The excitations to 3.94 eV and 4.92 eV, on the other hand (Figure 4b,d), originates mainly from $\pi_{-2}\to \pi_{0}^{*}(3.94\text{eV})$ and $\pi_{-6}\to \pi_{0}^{*}(4.92)$ transitions, where the *π*_−2_ and *π*_−6_ molecular orbitals are strongly localized at the imidazole group. The excitation energies of 4.04 eV is mainly assigned to a $\pi_{-4}\to \pi_{0}^{*}$ configuration (4.70 eV), with the *π*_−4_ wavefunction being spread across the whole extension of the benznidazole molecule. The 5.73 eV absorption peak, on the other hand, involves the HOMO (*π*_0_) orbital, mainly localized at the benzene and acetamide regions, and the $\pi_{+1}^{*}$ orbital, mainly localized at the benzene ring (Kohn-Sham orbital energy gap of 6.25 eV). Finally, the 5.87 eV transition is originated from a $\pi_{-4}\to \pi_{+3}^{*}$ configuration (7.74 eV), where the $\pi_{+3}^{*}$ state is mostly delocalized at the imidazole and acetamide groups.

In truth, benznidazole has low solubility in water (400 mg/L at 37 degrees Celsius), which precludes the preparation of liquid dosage forms and contributes to increase its toxicity. Several studies have been published proposing new forms to improve its solubility and, consequently, its bioavailability. For example, Lamas *et al.* [18] have investigated the cosolvation of BZN with ethyl alcohol, propylene glycol, polyethylene glycol (PEG) 400, benzyl alcohol, diethylene glycol monoethyl ether and some surfactants, obtaining formulations which remained stable for at least 1.5 years. A study on the release mechanism of BZN in solid dispersions with PEG 6000 and polyvinylpirrolydone K-30 (PVP K-30) [19] revealed a strong interaction between BZN and the polymers, especially for PVP, which has improved BZN solubility in water. Complexation of BZN with ruthenium has lead to a compound with increased activity and lower acute toxicity *in vitro* and *in vivo* [11]. It was shown that Hydroxypropyl-*β*-cyclodextrin (CD) produces a great improvement (20×) in the aqueous solubility of BZN, with solid systems prepaired with BNZ and CD exhibiting physical-chemical reactions which help to promote the dissolution rate [20]. Notwithstanding that, ordinary benznidazole administration is nowadays performed using 100 mg tablets which are rapidly absorbed by the gastrointestinal tract into the bloodstream, where the drug circulates in its pure form. As a matter of fact, our research group has performed biological essays testing the action of BZN on T. Cruzi using BZN concentrations in water similar to the values used to perform the optical absorption measurements presented in this work, which motivated us to consider only water as a solvent in the TD-DFT computations. It would be interesting to implement similar measurements and theoretical calculations for BZN in other solvents than water, and we are taking the first steps to carry out a more detailed study testing the effect of different solvation environments on the optical absorption spectrum of BZN (the results thus obtained will be published in the near future). In particular, preliminary data on the optical absorption of BZN in a solution of acetonitrile were shown to be almost identical to the case of BZN in aqueous solution, indicating that a solvation change does not necessarily modify the optical absorption by a significant amount.

## 4. Conclusions

In summary, we have presented the results of optical absorption measurements carried out for water solvated benznidazole (BNZ), the drug of choice for the treatment of Chagas disease, as well as the results of time-dependent density functional theory (TD-DFT) calculations to interpret them. An excellent agreement between theory and experiment data was achieved, with the measured absorption peaks being fitted by six Gaussian curves centered at 3.60, 3.83, 4.15, 4.99, 5.60, and 5.76 eV. The TD-DFT oscillator strengths predicted the smallest energy absorption bands at 3.87, 3.94, 4.04, 4.92, 5.73, and 5.87 eV. The main experimental absorption peak at 3.83 eV is assigned mainly to a $\pi_{-3}\to \pi_{0}^{*}$ transition involving an orbital delocalized along the benzene and acetamide regions and another one (LUMO) located mainly at the benzene ring of benznidazole. One conclude that the TD-DFT is able to reproduce very satisfactorily the experimental UV-vis optical absorption band shape of benznidazole in water. The results are useful for the characterization of benznidazole dissolved in water, an aid to the development of liquid benznidazole formulations mainly indicated for children and the elderly suffering with Chagas disease.

## Figures and Tables

**Table 1.** TD-DFT calculated states and electronic transitions which rule the UV-vis optical absorption of benznidazole in water (PCM model).

**State**	**E(eV)**	**Transition**	**Coeff**	**%**	**O Str**	**State**	**E(eV)**	**Transition**	**Coeff**	**%**	**O Str**
**1**	3.363	H-4 → L0	0.116	2.68	0.0013	**9**	4.921	H-7 → L0	0.110	2.43	0.0343
		H0 → L0	0.694	96.20				**H-6** → **L0**	**0.681**	**92.71**	

**2**	3.680	H-3 → L0	0.141	3.97	0.0022	**10**	5.262	H-1 → L+1	0.442	38.99	0.0010
		H-1 → L0	0.687	94.26				H-1 → L+2	0.150	4.52	
	
**3**	3.784	H-7 → L0	−0.307	18.85	0.0014			H0 → L+1	−0.205	8.42	
		H-5 → L0	0.628	78.97				H0 → L+2	0.476	45.35	

**4**	3.870	H-4 → L0	−0.313	19.62	0.0191	**11**	5.729	H-3 → L+1	0.114	2.59	0.0963
		**H-3** → **L0**	**0.580**	**67.38**				H-1 → L+1	0.119	2.85	
		H-2 → L0	−0.190	7.23				H-1 → L+2	−0.248	12.33	
		H-1 → L0	−0.165	5.46				**H0** → **L+1**	**0.564**	**63.72**	
	
**5**	3.935	H-4 → L0	−0.239	11.44	0.2396			H0 → L+2	0.222	9.82	
	
		**H-2** → **L0**	**0.654**	**85.49**		**12**	5.865	H-4 → L+2	0.234	10.90	0.0138
	
**6**	4.041	**H-4** → **L0**	**0.565**	**63.81**	0.0349			**H-4** → **L+3**	**0.302**	**18.22**	
		H-3 → L0	0.360	25.96				H-4 → L+4	−0.261	13.59	
		H-2 → L0	0.165	5.47				H-4 → L+5	−0.118	2.76	
		H0 → L0	0.128	3.30				H-3 → L+1	−0.116	2.71	
	
**7**	4.476	H-9 → L0	0.688	94.63	0.0013			H-3 → L+3	−0.139	3.88	
		H-8 → L0	−0.101	2.06				H-3 → L+4	0.117	2.73	
	
**8**	4.571	H-7 → L0	0.622	77.35	0.0011			H-1 → L+1	−0.145	4.18	
		H-6 → L0	−0.118	2.78				H0 → L+1	−0.171	5.87	
		H-5 → L0	0.309	19.09				H0 → L+3	0.266	14.10	
								H0 → L+4	0.216	9.34	

## Supplementary Materials

Supplementary materials can be accessed at: http://www.mdpi.com/1420-3049/19/4/4145/s1.

## References

[1] Coura J.T., de Castro S.L. (2002). A Critical Review on Chagas Disease Chemotherapy. Mem. Inst. Oswaldo Cruz..

[2] Hotez P.J., Molyneux D.H., Fenwick A., Kumaresan J., Sachs S.E., Sachs J.D., Savioli L. (2007). Control of Neglected Tropical Diseases. N. Engl. J. Med..

[3] Díaz-Urrutia C.A., Olea-Azar C.A., Zapata G.A., Lapier M., Mura F., Aguilera-Venegas B., Arán V.J., López-Múñoz R.A., Maya J.D. (2012). Biological and chemical study of fused tri- and tetracyclic indazoles and analogues with important antiparasitic activity. Spectrochim. Acta A..

[4] Salomon C.J. (2012). First century of Chagas' disease: An overview on novel approaches to nifurtimox and benznidazole delivery systems. J. Pharm. Sci..

[5] Durán N., Marcato P.D., Teixeira Z., Durán M., Costa F.T.M., Brocchi M. (2009). State of the Art of Nanobiotechnology Applications in Neglected Diseases. Curr. Nanosci..

[6] Romero E.L., Morilla M.J. (2010). Nanotechnological approaches against Chagas disease. Adv. Drug Deliv. Rev..

[7] Bern C. (2011). Antitrypanosomal Therapy for Chronic Chagas' Disease. N. Engl. J. Med..

[8] Soares-Sobrinho J.L., de La Roca Soares M.F., Lopes P.Q., Correia L.P., de Souza F.S., Macêdo R.O., Rolim-Neto P.J. (2010). A Preformulation Study of a New Medicine for Chagas Disease Treatment: Physicochemical Characterization, Thermal Stability, and Compatibility of Benznidazole. AAPS PharmSciTech..

[9] Rassi A., Marin-Neto J.A. (2010). Chagas Disease. Lancet.

[10] Nothenberg M.S., Takeda G.K., Najjar R. (1991). Adducts of nitroimidazole derivatives with rhodium(II) carboxylates: Syntheses, characterization, and evaluation of antichagasic activities. J. Inorg. Biochem..

[11] Silva J.J.N., Pavanelli W.R., Gutierrex F.R.S., Lima F.C.A., da Silva A.B.F., Silva J.S., Franco D.W. (2008). Complexation of the anti-Trypanosoma cruzi Drug Benznidazole Improves Solubility and Efficacy. J. Med. Chem..

[12] Soares-Sobrinho J.L., Cunha-Filho M.S.S., Rolim Neto P.J., Torres-Labandeira J.J., Dacunha-Marinho B. (2008). Benznidazole. Acta Cryst..

[13] Gunatilleke S.S., Calvet C.M., Johnston J.J., Chen C.-K., Erenburg G., Gut J., Engel J.C., Ang K.K.H., Mulvaney J., Chen S. (2012). Diverse Inhibitor Chemotypes Targeting Trypanosoma cruzi CYP51. PLoS Negl. Trop. Dis..

[14] Frisch M.J., Trucks G.W., Schlegel H.B., Scuseria G.E., Robb M.A., Cheeseman J.R., Scalmani G., Barone V., Mennucci B., Petersson G.A. (2009). Gaussian 09, Revision A.01.

[15] Marques M.A.L., Gross E.K.U. (2004). Time-Dependent Density Functional Theory. Annu. Rev. Phys. Chem..

[16] Casida M.E., Huix-Rotllant M. (2012). Progress in Time-Dependent Density-Functional Theory. Annu. Rev. Phys. Chem..

[17] Adamo C., Jacquemin D. (2013). The calculations of excited-state properties with Time-Dependent Density Functional Theory. Chem. Soc. Rev..

[18] Lamas M.C., Villagi L., Nocito I., Bassani G., Leonardi D., Pascutti F., Serra E., Salomón C.J. (2006). Development of Parenteral Formulations and Evaluation of the Biological Activity of the Trypanocide Drug Benznidazole. Int. J. Pharm..

[19] Lima A.A.N., Soares-Sobrinho J.L., Silva J.L., Corrêa-Júnior R.A., Lyra M.A., Santos F.L., Oliveira B.G., Hernandes M.Z., Rolim L.A., Rolim-Neto P.J. (2011). The Use of Solid Dispersion Systems in Hydrophilic Carriers to Increase Benznidazole Solubility. J. Pharm. Sci..

[20] Maximiano F.P., Costa G.H.Y., de Sá Barreto L.C., Bahia M.T., Cunha-Filho M.S. (2011). Development of Effervescent Tablets Containing Benznidazole Complexed with Cyclodextrin. J. Pharm. Pharmacol..

