# Supplementary material for: Optical Absorption of the Antitrypanocidal Drug Benznidazole in Water
Source: Molecules. 2014 Apr 2;19(4):4145–56. doi: 10.3390/molecules19044145 (PMC6271472; doi:10.3390/molecules19044145)
Supplement: Supplementary file 1 [file molecules-19-04145-s001.pdf]

Supplementary file

Table S1. Bond lenghts of benznidazole as obtained from X-ray diffraction (crystalline phase) and from the DFT calculated smallest conformer energy in water (PCM model).

Bond	EXP	THE	Bond	EXP	THE
C1-C2	1.391	1.396	N15-C16	1.314	1.317
C1-C6	1.388	1.397	C16-N17	1.435	1.432
C2-C3	1.383	1.393	N17-O18	1.226	1.235
C3-C4	1.384	1.397	N17-O19	1.232	1.227
C4-C5	1.391	1.393	N8-H27	0.836	1.011
C5-C6	1.396	1.401	C1-H20	0.950	1.085
C6-C7	1.505	1.516	C2-H21	0.950	1.084
C7-N8	1.468	1.463	C3-H22	0.950	1.084
N8-C9	1.329	1.349	C4-H23	0.950	1.084
C9-C10	1.528	1.539	C5-H24	0.950	1.085
C9-O11	1.228	1.226	C7-H25	0.990	1.091
C10-N12	1.463	1.460	C7-H26	0.990	1.093
N12-C13	1.358	1.364	C10-H28	0.990	1.088
N12-C16	1.362	1.373	C10-H29	0.990	1.091
C13-C14	1.370	1.383	C13-H30	0.950	1.078
C14-N15	1.362	1.357	C14-H31	0.950	1.078

Table S2. Bond angles of benznidazole as obtained from X-ray diffraction (crystalline phase) and from the DFT calculated smallest conformer energy in water (PCM model).

Degree	EXP	THE	Degree	EXP	THE	Degree	EXP	THE
C2-C1-C6	120.750	120.688	C6-C7-N8	110.860	112.813	H28-C10-H29	108.100	108.430
C2-C1-H20	119.600	119.726	C6-C7-H25	109.500	110.764	C10-N12-C13	124.170	124.302
C6-C1-H20	119.600	119.586	C6-C7-H26	109.500	109.818	C10-N12-C16	130.670	130.532
C1-C2-C3	119.740	120.060	N8-C7-H25	109.500	106.404	C13-N12-C16	104.990	105.109
C1-C2-H21	120.100	119.780	N8-C7-H26	109.500	109.328	N12-C13-C14	106.760	106.761
C3-C2-H21	120.100	120.159	H25-C7-H26	108.100	107.530	N12-C13-H30	126.600	121.541
C2-C3-C4	120.290	119.639	C7-N8-C9	121.090	123.773	C14-C13-H30	126.600	131.697
C2-C3-H22	119.900	120.216	C7-N8-H27	119.900	117.434	C13-C14-N15	110.690	110.135
C4-C3-H22	119.900	120.145	C9-N8-H27	118.200	118.764	C13-C14-H31	124.700	127.835
C3-C4-C5	119.860	120.172	N8-C9-C10	114.480	113.561	N15-C14-H31	124.700	122.030
C3-C4-H23	120.100	120.035	N8-C9-O11	124.460	124.805	C14-N15-C16	103.740	104.962
C5-C4-H23	120.100	119.793	C10-C9-O11	120.910	121.600	N12-C16-N15	113.810	113.031
C4-C5-C6	120.410	120.583	C9-C10-N12	110.810	111.742	N12-C16-N17	123.420	123.955
C4-C5-H24	119.800	119.705	C9-C10-H28	109.500	111.025	N15-C16-N17	122.740	123.012
C6-C5-H24	119.800	119.712	C9-C10-H29	109.500	108.800	C16-N17-018	118.300	117.774
C1-C6-C5	118.930	118.858	N12-C10-H28	109.500	109.718	C16-N17-O19	117.620	118.093
C1-C6-C7	120.410	120.319	N12-C10-H29	109.500	106.983	O18-N17-O19	124.070	124.133
C5-C6-C7	120.650	120.815						

Table S3. Dihedral angles of benznidazole as obtained from X-ray diffraction (crystalline phase) and from the DFT calculated smallest conformer energy in water (PCM model).

**Dihedral**	**EXP**	**THE**	**Dihedral**	**EXP**	**THE**
C7-N8-C9-O11	−1.00	−0.18	C10-N12-C13-C14	176.45	−177.98
C7-N8-C9-C10	−176.53	177.69	N15-C14-C13-N12	−0.46	0.27
C16-N15-C14-C13	0.00	0.06	C3-C4-C5-C6	0.00	−0.07
C2-C3-C4-C5	1.10	0.08	C1-C6-C5-C4	−1.10	−0.09
C4-C3-C2-C1	−1.10	0.07	C7-C6-C5-C4	179.30	178.85
C6-C1-C2-C3	0.00	−0.23	C14-N15-C16-N12	0.48	−0.39
C9-N8-C7-C6	167.75	−125.77	C14-N15-C16-N17	178.56	−179.94
C2-C1-C6-C5	1.10	0.24	C13-N12-C16-N15	−0.77	0.56
C2-C1-C6-C7	−179.29	−178.70	C10-N12-C16-N15	−176.13	−2.61
N8-C7-C6-C1	116.20	−125.10	C13-N12-C16-N17	−178.83	−179.89
N8-C7-C6-C5	−64.20	55.98	C10-N12-C16-N17	5.80	−2.61
C13-N12-C10-C9	−97.72	105.17	O18-N17-C16-N15	177.06	−179.76
C16-N12-C10-C9	76.86	−71.65	O19-N17-C16-N15	−3.80	0.20
O11-C9-C10-N12	20.40	−15.19	O18-N17-C16-N12	−5.00	0.74
N8-C9-C10-N12	−163.87	166.86	O19-N17-C16-N12	174.08	−179.31
C16-N12-C13-C14	0.70	−0.48			
